# Novel *TBC1D8B* variant causes neonatal nephrotic syndrome combined with acute kidney injury

**DOI:** 10.1186/s13052-024-01790-y

**Published:** 2024-10-29

**Authors:** Yuanyuan Xu, Chao Dai, Jing He, Yaping Liang, Ying Zhu, Fang Deng, Chang Wang, Danqun Jin

**Affiliations:** 1 Department of Pediatric Intensive Care Unit, Anhui Provincial Children’s Hospital, Children‘s Medical Center of Anhui Medical University, No. 39, Wangjiang Road, Hefei, 230051 Anhui China; 2 Department of Nephrology, Anhui Provincial Children’s Hospital, Hefei, 230051 China; 3 Department of Radiology, Anhui Provincial Children’s Hospital, Hefei, 230051 China

**Keywords:** Acute kidney injury, Nephrotic syndrome, TBC1D8B, Genetic testing

## Abstract

**Background:**

Nephrotic syndrome (NPHS), characterized by proteinuria, hypoalbuminemia, and edema, can be caused by genetic variations. *TBC1D8B* was recently discovered as a novel disease-causing gene for X-linked NPHS. With only a few reported cases, the clinical manifestations associated with variants of this gene need to be further examined.

**Methods:**

We recruited a newborn with NPHS complicated by acute kidney injury (AKI) and his parents and tested the potential genetic cause of the disease through trio-whole exome sequencing and Sanger sequencing. Western blotting (WB) was performed using a mutant plasmid to evaluate mutant protein expression levels. Since the TBC1D8B protein interacts with RAB proteins to catalyze the GTPase hydrolysis process, immunofluorescence (IF) can be used to verify the interaction between the TBC1D8B mutant protein and RAB11A/RAB11B, and thus to confirm its effect on the endocytosis and vesicle recycling functions of RAB proteins within the cell.

**Results:**

The child, at 1 month, showed severe edema and proteinuria and unexplained coma with epilepsy. Ultrasound examination revealed multiple organ enlargement, and MRI showed nonspecific high diffusion-weighted imaging signal characteristics in the splenium of the corpus callosum. Hematoxylin and eosin staining showed diffuse inflammatory cell infiltration in the renal interstitium and multifocal renal tubule lumen expansion. Diffuse fusion of podocyte foot processes was observed under electron microscopy, indicating glomerular podocyte lesions. Genetic testing revealed a maternally inherited novel hemizygous variant, NM_017752: c.628 A > T, p.Lys210Ter, in *TBC1D8B*. In vitro functional experiments showed that this variant may lead to TBC1D8B protein degradation. IF results showed disrupted interaction with RAB11A/RAB11B, that then affects the biological function of RAB proteins in the process of cell intimal vesicle formation and intracellular transport.

**Conclusion:**

This study will enrich the mutational and phenotypic spectra of *TBC1D8B* and demonstrate the potential of this gene variants to cause early-onset NPHS leading to severe kidney disease.

**Supplementary Information:**

The online version contains supplementary material available at 10.1186/s13052-024-01790-y.

## Background

Nephrotic syndrome (NPHS) is a common renal disease in children. It manifests mainly as the clinical triad of proteinuria, hypoalbuminemia, and varying degrees of edema in the nephrotic range [1, 2]. The occurrence of NPHS is related to glomerular filtration function, in which damage to glomerular podocytes (also called visceral epithelial cells), such as podocyte foot process fusion, disappearance, or apoptosis, is a common pathological feature [3, 4]. The pathogenesis of NPHS is complex. Most pediatric cases may be related to autoimmune dysfunction, some cases may be caused by congenital factors, and more than 50% of NPHS cases in infants (< 12 months old) have genetic causes [5, 6]. Currently, more than 50 genes have been identified to be related to podocyte-related pathologies and are involved in maintaining the stability of the podocyte skeleton or slit diaphragm (SD) structures [7]. Dorval et al. first reported a *TBC1D8B* variation in two families with NPHS [8]. This gene encodes a protein with a Tre-2/Bub2/CDC16 (TBC) domain, which can activate GTPase-activating protein (GAP) and accelerate GTPase hydrolysis. It also participates in endosomal trafficking among podocytes [8]. This mechanism is relatively rare in monogenic NPHS, and only 13 patients with NPHS-related *TBC1D8B* variants have been reported in five studies [8–12]. However, these patients generally develop symptoms from childhood to adulthood, and only one case has been reported in a 6-month-old infant [10]. Therefore, there is a lack of research on the clinical manifestations and mechanisms of *TBC1D8B* defects in early life stages.

We assessed clinical reports and conducted genetic analyses of patients with neonatal NPHS and acute kidney injury (AKI). Genetic testing identified a novel variant of *TBC1D8B*, and in vitro functional studies further demonstrated its pathogenicity. This study aimed to enrich our understanding of the phenotypic spectrum of *TBC1D8B* defects and to reveal the potential mechanisms and key roles of this novel variant in kidney development.

## Materials and methods

### Human subjects

All the procedures were performed in accordance with the ethical standards of the 1975 Declaration of Helsinki (2000 revised edition). The disclosure of clinical data, including imaging and variation data, was performed with informed consent from the subject’s legal guardian. This study was approved by the Ethics Committee of Anhui Provincial Children’s Hospital, Anhui, China (Ethical approval number: EYLL-2021-024).

### Gene testing

Genomic DNA was extracted from the peripheral blood of the proband and his parents for trio whole-exome sequencing (trio-WES). The exomes of the samples were captured using the xGen Exome Research Panel v2.0 (IDT, IA, USA). Raw sequencing data were obtained using an Illumina NovaSeq 6000 series (Illumina, San Diego, CA, USA) for paired-end reads. After removing adapters and low-quality reads, Burrows–Wheeler alignment software was used to compare with the GRCh37/hg19 sequences. The GATK software was used to call the SNPs and indel data. The variant population distribution frequency databases used in this study included gnomAD, dbSNP, ExAC, and 1000 Genomes. The online platforms SIFT, Polyphen2, REVEL, and MutationTaster were used to predict the impact of mutations on protein product structure. Finally, the pathogenicity of the variants was annotated according to the American College of Medical Genetics and Genomics (ACMG) criteria and guidelines [13].

### Sanger sequencing

Sanger sequencing was performed on exon 5 and its flanking region (*TBC1D8B*, NM_017752.3) in the proband and his family members. The polymerase chain reaction (PCR) product was amplified using the primers F: 5’-TAGTTTGCCAGCTGTTCCATAAGA-3’ and R: 5’-ACCAATTCTTGCACGTTCATGTTG-3’. The products were purified using a PCR Purification Kit (Qiagen) and sequenced using an ABI 3730XL DNA Analyzer (Applied Biosystems).

### TBC1D8B cDNA constructs

The wild-type TBC1D8B construct was cloned into the pECMV-3×FLAG-N vector (Flag-tagged TBC1D8B-WT) using the Phanta Max Super-Fidelity DNA Polymerase kit (#P505, Vazyme, Xuanwu Qu, China), following the manufacturer’s instructions. The amplification primers were (F) 5’-cttggtaccgagctcggatccATGTGGCTGAAGCCTGAGGA-3’ and (R) 5’-tgctggatatctgcagaattcCTACTTCTCCCCAATAACACATTGTC-3’. The mutant plasmid (FLAG-tagged TBC1D8B-Lys210Ter) was generated using the site-directed mutagenesis kit V2 (#C215-01, Vazyme). The primer designs are shown in Supplementary Table S1. Plasmids were purified using a QIAGEN Plasmid Mini Kit (QIAGEN, Limburg, Germany) and PureLink HiPure Plasmid Midiprep Kit (Life Technologies, CA, USA). All plasmid construct sequences were confirmed by Sanger sequencing.

### Cell culture and plasmid transfection

Human 293T cells (Shanghai Cell Bank) were cultured in high-glucose DMEM (Thermo Fisher Scientific, Waltham, MA, USA) containing 10% fetal calf serum and 1% penicillin. The incubator environment was 5% CO_2_ at 37 ℃. Plasmid transfection was performed using the Lipofectamine 3000 reagent (Thermo Fisher Scientific). After transfection, the cells were lysed using a protein lysis buffer (#P0013, Beyotime, China), and the proteins were extracted for subsequent experiments.

### Western blot (WB)

The cell lysate was subjected to protein separation by 10% sodium dodecyl sulfate-polyacrylamide gel electrophoresis, and the proteins were electrotransferred to a polyvinylidene difluoride-plus transfer membrane. WB was performed using DYKDDDDKTag (9A3) Mouse mAb (Cat#8146) and β-Actin (D4C6R) Mouse mAb (Cat#3700) at 1:1000 (Cell Signaling Technology, Danvers, MA, USA) and incubated overnight at 4 °C. The membrane was then incubated with HRP-linked anti-mouse IgG secondary antibody (Cat#7076, dilution ratio 1:5000, Cell Signaling Technology). Clarity Western ECL Substrate (Bio-Rad Laboratories, Hercules, CA, USA) was used for protein development. Image J v1.46 software (NIH, Bethesda, MD, USA) was used to analyze WB bands.

### Immunofluorescence (IF)

293T cells were transfected with Flag-TBC1D8B-WT/Flag-TBC1D8B-Lys210Ter and MyC-RAB11/MyC-RAB11B. Transfected cells were passaged in confocal dishes (#BS-20-GJM; Biosharp, Hefei, China). The cells were fixed with 4% paraformaldehyde tissue fixative (#P0099; Beyotime, Shanghai, China) for 15 min and permeabilized with 0.5% Triton X-100 (#P0096) at room temperature for 15 min. The cells were incubated overnight at 4 °C with DYKDDDDKTag (9A3) Mouse mAb (Cat#8146) and Myc-Tag (71D10) Rabbit mAb (Cat#2278) at a dilution ratio of 1:200 (Cell Signaling Technology). This was incubated with the CoraLite488–conjugated goat anti-Mouse IgG (H + L; Cat No. SA00013-1, dilution ratio 1:100, Sanying, Wuhan, China) for 1 h at 37 °C. After washing with phosphate-buffered solution (PBS), the appropriate amount of DAPI staining solution (#C1005, Beyotime, Shanghai, China) was added and incubated in the dark for 5 min. The excess staining solution was washed away with PBS, and images were taken under a confocal microscope (Leica TCS SP8, Germany).

### Statistical analysis

GraphPad Prism 8 (GraphPad Software, CA, USA) was used to perform t-test and draw graphs. Statistical significance was set at *P* < 0.05. Experiments were performed in triplicate.

## Results

### Clinical description

The 1-month-old male infant was admitted to the Pediatric Intensive Care Unit of Anhui Children’s Hospital in an unknown “coma” state. He was the second child of nonconsanguineous parents. The child’s parents and 4-year-old sister were both in good health and denied family genetic history or incest. The routine urine test results of family members were normal. The child was delivered vaginally at 41 weeks with a birth weight of 3600 g. His mother denied any history of asphyxia at birth. Notably, the Apgar score at 1 min was 10, and at 5 min was 10. The child developed reduced urine output on the 27th day after birth and suddenly manifested an unexplained coma with a Glasgow Coma Scale score of 6 (Eye opening 1, Verbal response 1, Motor response 4). Physical examination on admission showed a pale complexion, cyanosis of the lips, moderate edema throughout the body, moderate edema on both eyelids, poor reaction ability, equal-sized and round pupils on both sides, and a reflex reaction to light. After admission, the patient was placed on invasive mechanical ventilation with pressure-regulated volume control mode (settings: fraction of inspired oxygen was 40%, tidal volume was 60 mL, respiratory rate was 30 breaths/minute, positive end-expiratory pressure was 4 cmH_2_O) to assist with breathing. On the second day after admission, the patient had seizures characterized by staring eyes, cyanotic lips, hypertonia in limbs, and fecal incontinence.

**Fig. 1:**
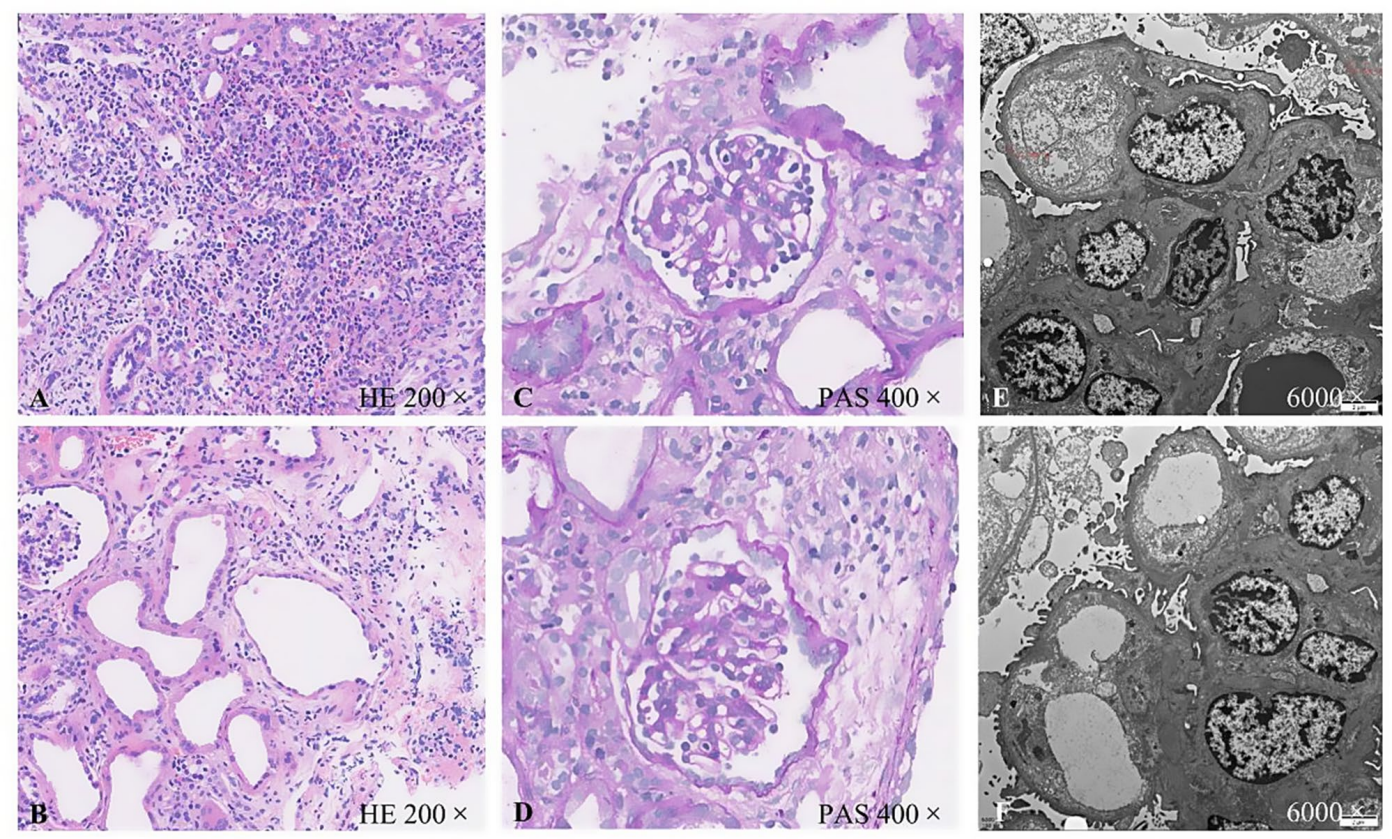
Staining and ultrastructure of pathological sections of this neonate. **A** Diffuse inflammatory cell infiltration in the renal interstitium (mainly lymphocytes and monocytes, with focal eosinophils and neutrophils infiltration) seen under a light microscope; **B** Multifocal renal tubule lumen dilation, with some tubules showing cystic dilation; **C**,** D.** Poorly developed and cube-shaped glomerular podocytes seen under light microscopy. The glomerular capillary loops are well opened, without endothelial cell proliferation and occasional inflammatory cell infiltration. Focal segmental mild hyperplasia of mesangial cells and stroma in the glomerular mesangial zone can also be observed; **E**,** F.** Diffuse fusion of podocyte foot processes, accompanied by microvilliation, as seen under electron microscopy (the fusion area is about 80%)

Laboratory tests showed reduced red blood cell, hemoglobin, and platelet counts, with slightly higher reticulocytes. Biochemical examination showed reduced total protein, albumin, and globulin levels; disturbed electrolytes (blood sodium and blood chloride levels were decreased); and increased serum creatinine. Urinalysis showed abnormally high levels of total protein and microprotein (Table 1). Serum creatinine reached 278.7 umol/L, exceeding the baseline by more than three times (given the patient was only 1 month old), and urine output was < 20 mL in 26 h after admission, < 0.3 mL/(kg·h). According to the Kidney Disease: Improving Global Outcomes (KDIGO) criteria, this met the diagnosis of AKI stage 3 [14]. To further clarify the pathological characteristics of NPHS and AKI, pathological examination of the right kidney revealed glomerular podocyte lesions and acute tubulointerstitial injury. Ultrastructurally, diffuse fusion of the foot processes of the glomerular podocytes was observed (Fig. 1).

**Table 1:** Data from the patient’s initial laboratory examination

Item, unit	Value	Reference
Peripheral blood routine examination		
white blood cell, 10^9^/L	10.24	5.6–14.5
hemoglobin, g/L	57 ↓	99–196
platelets, 10^9^/L	96 ↓	203–653
reticulocyte percentage, %	2.62↑	0.82–2.25
mean corpuscular volume, fL	99.40	73.00-105.00
total cholesterol, mmol/L	0.94	0-5.20
biochemical examination		
total bilirubin, µmol/L	20.4 ↑	2.0–21.0
direct bilirubin, µmol/L	18.4 ↑	0–8
indirect bilirubin, µmol/L	2.0	2–18
total protein, g/L	38.8 ↓	49–71
albumin, g/L	30.8 ↓	35–50
globulin, g/L	8.0 ↓	9–27
alanine aminotransferase, U/L	828.0 ↑	8–71
aspartate aminotransferase, U/L	917.0 ↑	21–80
urea, mmol/L	24.9 ↑	0.8–5.3
creatinine, µmol/L	278.7 ↑	13–33
uric acid, µmol/L	1020.4 ↑	208–428
blood potassium, mmol/L	5.79	4.2–5.9
blood sodium, mmol/L	114 ↓	135–150
blood chloride, mmol/L	81 ↓	100–116
plasma osmolality, mOsm/kg•H	248,74 ↓	275–300
urine test		
microalbumin, mg/L	9871.8 ↑	0–20.0
urine total protein, mg/L	13154.0 ↑	0-120.0
urinary retinol binding protein, mg/L	34.9 ↑	0-0.7
urinary transferrin, mg/L	389.80 ↑	0.00–2.00
urinary β2 microglobulin, mg/L	25.26 ↑	≤ 0.30
N-acetyl-β-D-glucosaminidase, U/L	14.6 ↑	< 12.0

**Fig. 2:**
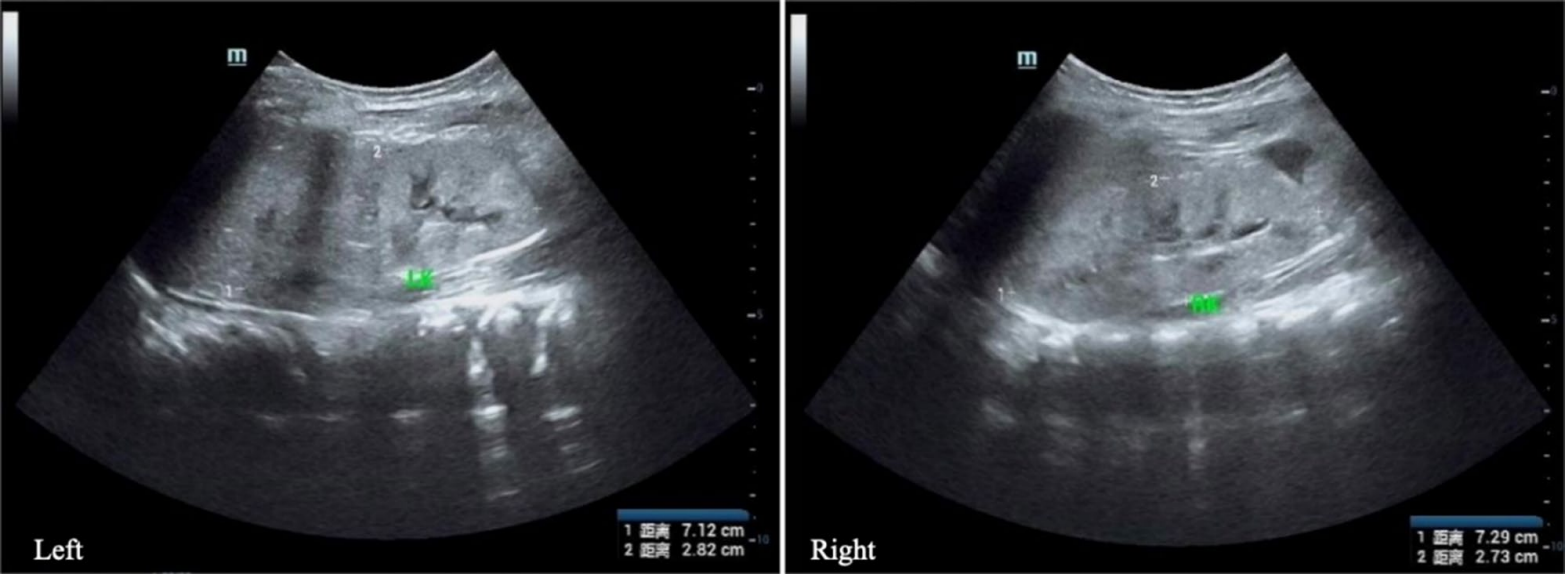
Ultrasound examination of the kidneys. Ultrasound examination shows that the size of the left kidney is about 7.1 × 2.8 cm, and the size of the right kidney is about 7.3 × 2.7 cm. The parenchymal echo of both kidneys is enhanced, and the boundary between the upper cortex and medulla of the right kidney is unclear

**Fig. 3:**
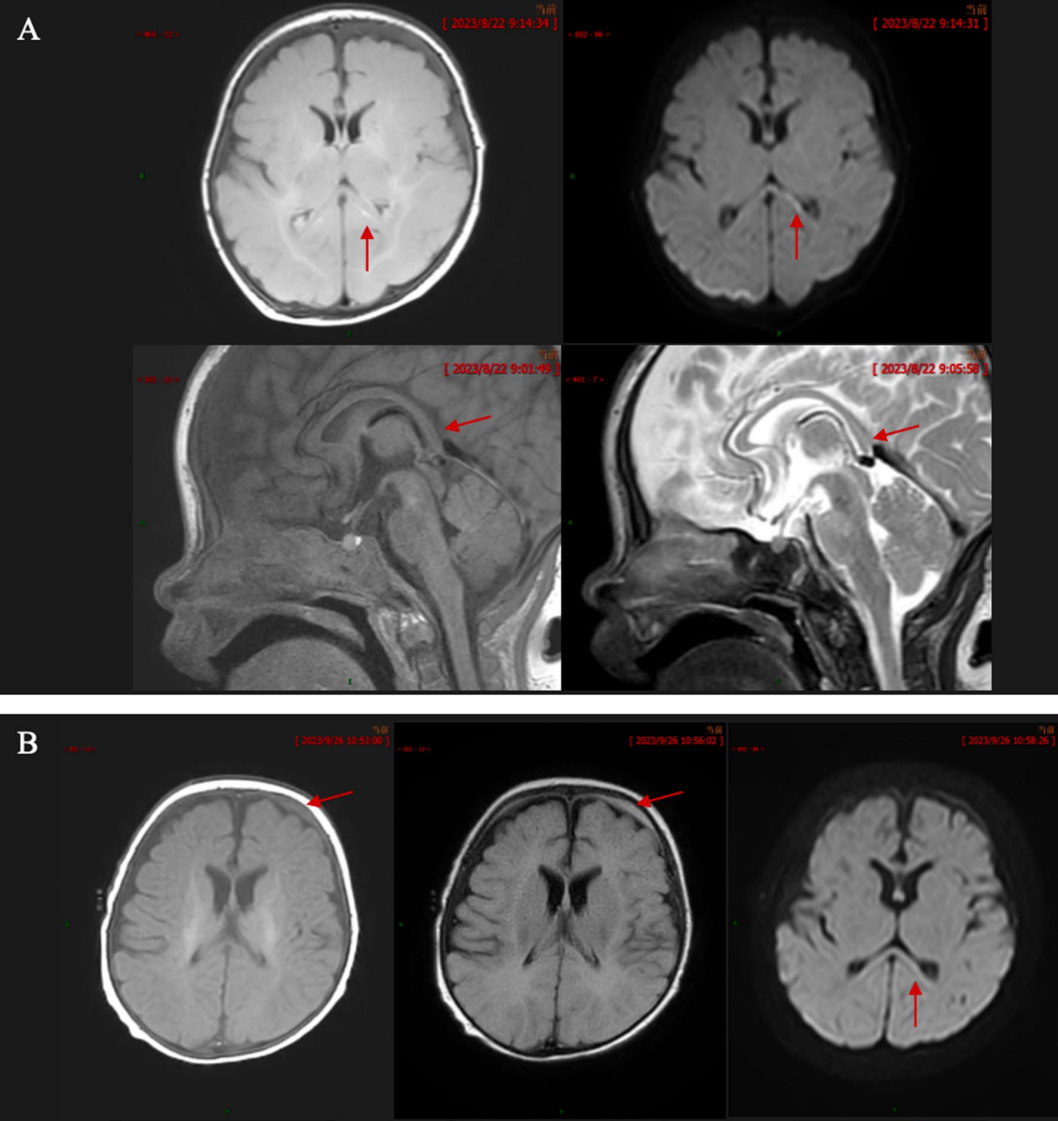
Cephalic MRI. **A** MRI examination on the 4th day after admission. The FLAIR sequence on the left side of the splenium of the corpus callosum showed a slight thickening, and a strip of high signal can be seen behind the splenium; DWI shows a high signal; T1 sagittal view shows a strip of low signal on the splenium of the corpus callosum. The T2 sagittal view shows high signal intensity in the splenium of corpus callosum; **B** MRI reexamination after 5 weeks of treatment. A strip of T1 signal is seen under the left frontal skull plate, the local signal in the FLAIR sequence is slightly higher, and the high signal in the splenium of the corpus callosum disappeared on DWI

The child’s proteinuria resulted in hypoalbuminemia and decreased plasma colloid osmotic pressure, leading to generalized edema, and the development of ascites and pelvic fluid extravasation. Abdominal ultrasound revealed diffuse renal lesions, ascites, pelvic fluid, and hepatosplenomegaly (Fig. 2). Lung CT showed exudative lesions in both lungs, bilateral pleural effusion, and bronchial narrowing with flocculent high-density shadows. The subcutaneous soft tissue edema in the neck, bilateral armpits, chest, and back was considered linked with hypotonic hypervolemic hyponatremia-hypochloremia, based on the biochemical data. Head magnetic resonance imaging (MRI) showed that the fluid attenuated inversion recovery (FLAIR) sequence on the left side of the splenium of the corpus callosum showed slight thickening, and a strip of high signal could be seen behind the splenium (Fig. 3). Other special examinations included video electroencephalography, which documented sharp and slow waves in the left rolandic area. Electrocardiography revealed normal findings.

### Variant analysis

**Fig. 4:**
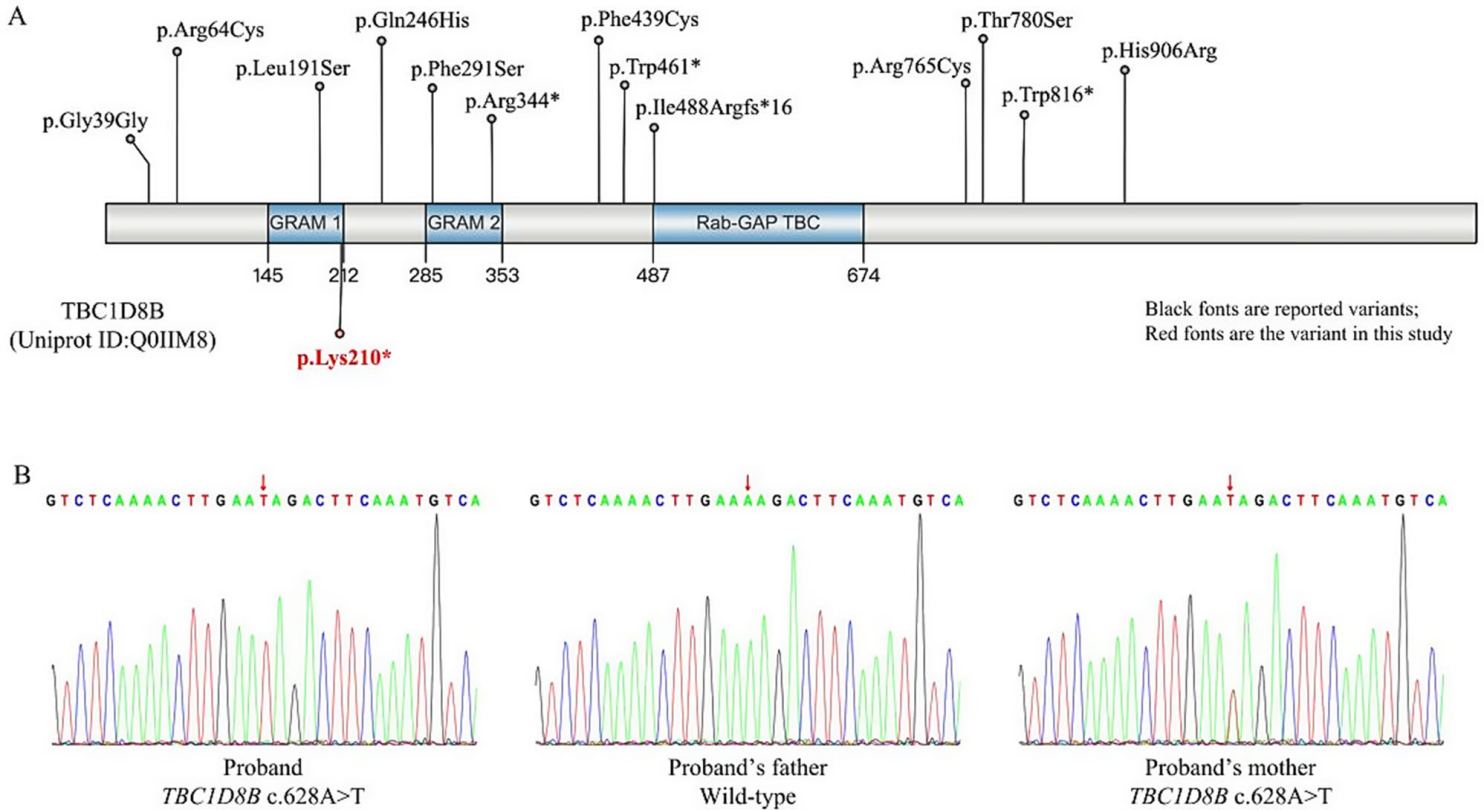
Genetic variation analysis. **A** The TBC1D8B protein mainly consists of two glucosyl transferases (GRAM) and one Tre2-Bub2-Cdc16 (TBC) domain. So far, 13 variants have been reported, and the novel variant identified in this study is located in the GRAM1 domain (marked in red); **B** Sanger sequencing of the proband. The sequencing confirmed that the proband had a hemizygous variant (NM_017752: c.628 A > T, p.Lys210Ter) in the *TBC1D8B*. The variant was inherited from his mother, and his father had the wild-type variant

Trio-WES revealed a *TBC1D8B* variant (NM_017752: c.628 A > T, p.Lys210Ter) located on chromosome Xq22.3, causing Lys 210 transition into a termination codon, and the protein to terminate translation at amino acid 210. The variant was inherited from the proband’s mother; the father’s was the wild type. Both parents had no clinical features related to kidney disease, which was consistent with the genetic co-separation. This variant is novel and has not been previously reported. According to the ACMG guidelines, this variant is rated as pathogenic. Furthermore, Sanger sequencing confirmed the presence of this variant (Fig. 4).

### In vitro functional experiments

**Fig. 5:**
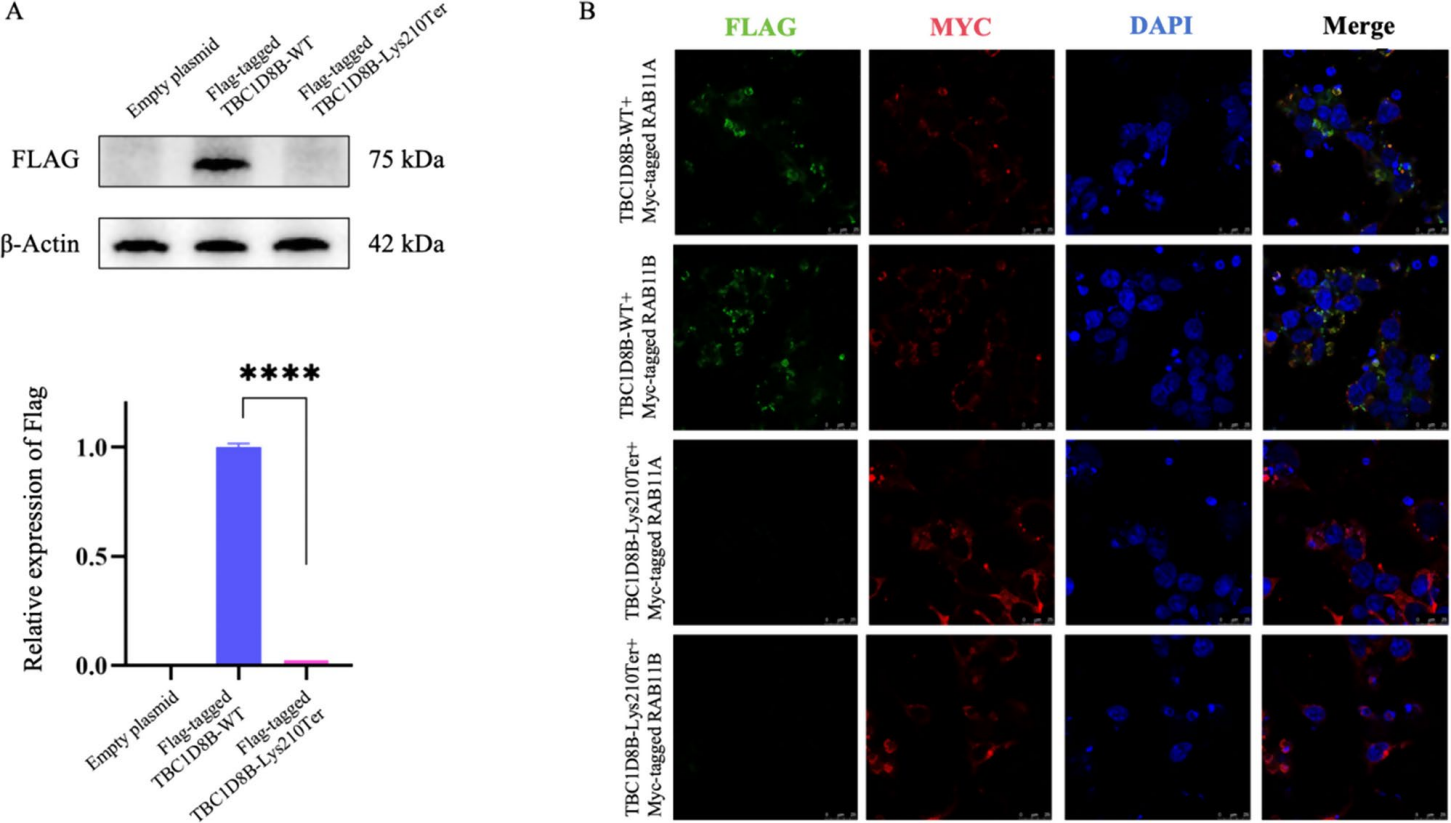
Variant functional analysis. **A** Western blot results for wild-type plasmid (Flag-tagged TBC1D8B-WT) and the mutant plasmid (Flag-tagged TBC1D8B-Lys210Ter). The protein expression level of the wild-type plasmid is significantly higher than that of the mutant plasmid. ****, *P* < 0.0001; **B** Immunofluorescence in the 293T cells transfected with the wild-type and mutant plasmids. Flag-tagged TBC1D8B-WT can bind to Myc-tagged RAB11A/Myc-tagged RAB11B and distribute in the cytoplasm. However, there is almost no fluorescence intensity of the mutant protein in Flag-tagged TBC1D8B-Lys210Ter + Mys-tagged RAB11A and Flag-tagged TBC1D8B-Lys210Ter + Mys-tagged RAB11B. This suggests that the variant may lead to protein degradation

Western blotting suggested that the mutant protein, owing to an early termination codon, may undergo degradation, limiting its expression. Expression levels of FLAG-tagged TBC1D8B-WT were significantly higher than FLAG-tagged TBC1D8B-Lys210Ter, which showed no difference compared to the empty vector plasmid. IF indicated that FLAG-tagged TBC1D8B-Lys210Ter had lower fluorescence intensities with Myc-tagged RAB11A/RAB11B compared with controls, suggesting impaired interaction with RAB11A/RAB11B. These results collectively indicate that the variant may lead to protein degradation and disrupt its interaction with RAB11A/RAB11B (Fig. 5).

## Discussion

In this study, we conducted a genetic analysis of a neonatal patient with NPHS who presented with severe edema and massive proteinuria. Genetic testing revealed a hemizygous variant in *TBC1D8B*. In vitro functional experiments showed that this variant results in almost no protein expression and disrupts interactions with RAB11A and RAB11B proteins. This may interfere with GAP function and affect endosomal trafficking of the SD protein nephrin, causing NPHS [9]. The patient reported in this study developed NPHS and AKI during the neonatal period, along with coma and epileptic symptoms. At the same time, because of hypoproteinemia, the plasma colloid osmotic pressure was reduced, resulting in systemic edema and abdominal and pelvic effusion. Brain MRI showed a high DWI signal in the splenium of the corpus callosum, which disappeared after 5 weeks of continuous renal replacement therapy. This may have been because of the reversible splenial lesion syndrome of the corpus callosum caused by hyponatremia. In addition, a diffuse fusion of the patient’s podocyte foot processes was observed in the ultrastructure of the pathological tissue, which was consistent with previous reports [8, 11]. However, the pathological staining analysis of multiple cases previously reported showed focal segmental glomerulosclerosis (FSGS), but our patient did not have FSGS features and showed renal tubular and interstitial damage, which may be related to the occurrence of AKI. This patient’s onset at such a young age, and with multisystem involvement, is the first report of TBC1D8B-related NPHS, which expands the genomic and phenotypic spectrum of this syndrome.

The protein encoded by *TBC1D8B* is a member of the Tre2-Bub2-Cdc16 (TBC) protein family, containing 1120 amino acids, consisting of two gucosyl transferases (GRAM) and a TBC domain [8]. GRAM can bind to lipid rafts and is a key component of SD signaling in podocytes [15]. The TBC domain comprises approximately 200 amino acid residues. This structure provides two key transcatalytic residues, arginine and glutamine, to form a dual-finger similar to that stabilizing the RAB protein in the transition state and catalyzes the GTPase hydrolysis process of the RAB protein [16]. The novel variant of *TBC1D8B* identified in this study resulted in the premature termination of the encoded amino acid and loss of the TBC domain and part of the GRAM structure. In vitro experiments showed that this variant may lead to protein degradation.

RAB is an evolutionarily conserved small GTPase in eukaryotic cells primarily involved in intracellular membrane vesicle formation and transport processes [17]. Kampf et al. showed that TBC1D8B can specifically bind to RAB11A and RAB11B, indicating that RAB11 has GAP activity [9]. We also verified through IF experiments that the identified mutant protein disrupted its interaction with the RAB11 protein. This indicates that the loss of function of TBC1D8B may affect the biological function of RAB11 as a GAP, thereby reducing cellular endocytosis and vesicle recycling.

Although *TBC1D8B* plays a unique and rare role in the pathogenesis of NPHS, its clinical phenotype does not differ from that of other NPHS types [7]. The OMIM database maps *TBC1D8B* defects to NPHS20 (OMIM#301028). To date, only 13 cases of nephrotic syndrome type 20 (NPHS20) have been reported. Most patients (10/13, 77%) had albuminuria from childhood (3–9 years old) to adulthood (18–59 years old) [8–12]. Only three children had the first onset of the disease under 3 years of age, and the clinical features of younger children seemed to be more severe. The 13 reported *TBC1D8B* variants were mainly missense variants (9/13, 69%); three nonsense variants and one frameshift variant were also reported, some of which were located in key structural domains (TBC and GRAM). However, these variants did not appear to be associated with the phenotypic severity of the disease, but conclusive data are not available owing to a limited number of reports.

With the wide application of next-generation sequencing (NGS) technology, an increasing number of cases have been revealed to be related to genetic variation. However, the phenotypes of some patients diagnosed with genetic variation are not consistent with those previously reported, which has led to confusion in clinical diagnosis or genetic counseling [18–20]. For example, the patient in this study showed the reported characteristics of NPHS, podocyte foot process diffuse fusion, and epilepsy. However, its early onset and the presence of unreported AKI manifestations make us believe that *TBC1D8B* variant may have other pathological mechanisms or phenotypes. Therefore, further case reports will be conducive to the understanding of the disease. In addition, genetic diagnosis also provides ideas for gene therapy and personalized care. Newborns and children with NPHS need multidisciplinary joint management and follow-up, involving neonatologists, nephrologists, neurologists, and geneticists. Careful genetic counseling with the parents of the child will help to avoid the risk of fetal disease during reproduction, while also providing pyschological and emotional support throughout the consultation process to cope with potential health management challenges [20, 21].

There are some limitations to this study. First, we failed to conduct a long-term clinical follow-up of the child, so we were unable to obtain more clinical information about the development of nephropathy and nervous system development. Second, there was a lack of validation of nephrin’s trafficking pathways in the experimental design.

## Conclusions

We report a rare case of a neonatal patient with a novel variant of the *TBC1D8B* gene, expanding the genomic and phenotypic spectrum of NPHS20. In vitro functional studies showed that the variant could significantly reduce the protein expression level and affect the interaction with RAB11A/RAB11B protein. We compiled clinical reports and research on NPHS20 in the early stages of life and showed the important value of genetic testing in neonatal nephropathy.

## Electronic supplementary material

Below is the link to the electronic supplementary material.

Supplementary Material 1

## Data Availability

The data underlying this article are available in the article and its supplementary material.

## Abbreviations

NPHS: Nephrotic syndrome
SD: Slit diaphragm
TBC: Tre-2/Bub2/CDC16
GAP: GTPase-activating protein
AKI: Acute kidney injury
WB: Western blot
IF: Immunofluorescence
MRI: Magnetic resonance imaging
FSGS: Focal segmental glomerulosclerosis
GRAM: Glucosyl transferases
