# Supplementary material for: Novel TBC1D8B variant causes neonatal nephrotic syndrome combined with acute kidney injury
Source: Ital J Pediatr. 2024 Oct 29;50:222. doi: 10.1186/s13052-024-01790-y (PMC11520770; doi:10.1186/s13052-024-01790-y)
Supplement: Supplementary file 1 — Supplementary Material 1 [file 13052_2024_1790_MOESM1_ESM.docx]

**Supplementary Table S1**

| Primer target | Primer sequence |
| --- | --- |
| TBC1D8B-F | 5’-cttggtaccgagctcggatccATGTGGCTGAAGCCTGAGGA-3’ |
| TBC1D8B-T-R | 5’-TGAAGTCTaTTCAAGTTTTGAGACTTCATCCCA-3’ |
| TBC1D8B-T-F | 5’-AACTTGAAtAGACTTCAAATGTCATACTGACAGAGAG-3’ |
| TBC1D8B-R | 5’-tgctggatatctgcagaattcCTACTTCTCCCCAATAACACATTGTC-3’ |
